# Validation of the Chinese Version of the Quality of Nursing Work Life Scale

**DOI:** 10.1371/journal.pone.0121150

**Published:** 2015-05-07

**Authors:** Xia Fu, Jiajia Xu, Li Song, Hua Li, Jing Wang, Xiaohua Wu, Yani Hu, Lijun Wei, Lingling Gao, Qiyi Wang, Zhanyi Lin, Huigen Huang

**Affiliations:** 1 Department of Nephrology, Guangdong General Hospital and Guangdong Academy of Medical Sciences, Guangzhou, China; 2 Cardiovascular Department, Gaomi People’s Hospital, Gaomi, China; 3 Nursing Department, Guangdong General Hospital and Guangdong Academy of Medical Sciences, Guangzhou, China; 4 School of Nursing, University of Texas Health Science Center at Houston, Houston, Texas, United States of America; 5 School of Nursing, Sun Yat-sen University, Guangzhou, China; 6 Digestive Department, Guangdong General Hospital and Guangdong Academy of Medical Sciences, Guangzhou, China; 7 Institute of Geriatric Medicine, Guangdong General Hospital and Guangdong Academy of Medical Sciences, Guangzhou, China; Kuopio University Hospital, FINLAND

## Abstract

Quality of Nursing Work Life (QNWL) serves as a predictor of a nurse’s intent to leave and hospital nurse turnover. However, QNWL measurement tools that have been validated for use in China are lacking. The present study evaluated the construct validity of the QNWL scale in China. A cross-sectional study was conducted conveniently from June 2012 to January 2013 at five hospitals in Guangzhou, which employ 1938 nurses. The participants were asked to complete the QNWL scale and the World Health Organization Quality of Life abbreviated version (WHOQOL-BREF). A total of 1922 nurses provided the final data used for analyses. Sixty-five nurses from the first investigated division were re-measured two weeks later to assess the test-retest reliability of the scale. The internal consistency reliability of the QNWL scale was assessed using Cronbach’s α. Test-retest reliability was assessed using the intra-class correlation coefficient (ICC). Criterion-relation validity was assessed using the correlation of the total scores of the QNWL and the WHOQOL-BREF. Construct validity was assessed with the following indices: χ^2^ statistics and degrees of freedom; relative mean square error of approximation (RMSEA); the Akaike information criterion (AIC); the consistent Akaike information criterion (CAIC); the goodness-of-fit index (GFI); the adjusted goodness of fit index; and the comparative fit index (CFI). The findings demonstrated high internal consistency (Cronbach’s α = 0.912) and test-retest reliability (interclass correlation coefficient = 0.74) for the QNWL scale. The chi-square test (χ^2^ = 13879.60, df [degree of freedom] = 813 P = 0.0001) was significant. The RMSEA value was 0.091, and AIC = 1806.00, CAIC = 7730.69, CFI = 0.93, and GFI = 0.74. The correlation coefficient between the QNWL total scores and the WHOQOL-BREF total scores was 0.605 (p<0.01). The QNWL scale was reliable and valid in Chinese-speaking nurses and could be used as a clinical and research instrument for measuring work-related factors among nurses in China.

## Introduction

Widespread nursing shortages and high nursing turnover have become global issues [1–3]. In America, a survey of 1793 nurses employed at 69 hospitals indicated that 67.5% of nurses reported the intention to leave within the next 1 to 3 years, whereas the percentage of nurses planning to leave the profession was 29.4% [4]. In China, the situation is also serious. China has a population of 1.3 billion and approximately 2.18 million nurses [5], and 12.8% of nurses leave their jobs in mainland China every year [6].

There are many reasons that nurses leave their jobs in China, including nursing professionals being a highly mobile workforce. With ongoing healthcare reforms and greater job mobility, mainland China faces the dual challenges of experiencing nurse resource difficulties and increasing nurse migration to more developed countries [7]. The practice environment has been associated with retention and job satisfaction, with a good-quality practice environment predicting better job satisfaction and increased nurse retention [8]. In China, a cross-sectional study of 21 hospitals reported that more than 50% of nurses were dissatisfied with their jobs, and a better work environment for nurses was associated with decreased job dissatisfaction and job-related burnout [9]. Thus, the identification of methods to increase nurses’ quality of nursing work life (QNWL) and job satisfaction is necessary to maintain a healthy and sustainable nursing workforce in China.

Quality of work life (QWL) is a complex, multidimensional concept that has been defined in different ways by different researchers [10]. However, a clear definition of QWL remains lacking. QWL refers to the employee’s satisfaction with his or her working life. Improving an employee’s QWL will improve the organization’s productivity and the employee’s self-actualization. The outcomes of QWL in nursing include nurses’ satisfaction, stress, burnout, retention, client satisfaction, and the quality of care [11].The QNWL is the degree to which registered nurses are able to satisfy important personal needs through their experiences in their work organization while achieving the organization’s goals [12]. QNWL is a comprehensive concept including various aspects of work itself and the work environment. Given that QNWL can be influenced by various factors, scholars and organizations have focused attention on how to scientifically assess the work conditions and mental statuses of nurses.

The World Health Organization developed an instrument in 1996 that is used globally to assess QNWL [13]. However, this instrument is not specialized to assess the quality of life for people from all countries. In 2005, Brooks and Anderson developed the QNWL scale [12]. They examined the modified scale using a sample of 1500 registered nurses (RNs) in a Midwestern state.

Currently, no validated instrument is available to evaluate the QNWL in China. Therefore, it is necessary to develop a cultural scale to measure the quality of nursing work life. By translating the established international scales and considering political, social, and cultural differences, we developed a new scale to assess the quality of nursing work life in China and to evaluate the construct validity of the QNWL scale.

## Methods

### Samples

A cross-sectional study was conducted conveniently from June 2012 to January 2013 at five hospitals in Guangzhou, which employ 1938 nurses. While some researchers have suggested that a sample size of 200 was adequate for factor analysis [14], others have recommended a sample of 5 or more individuals per item as an adequate size for factor analysis [15]. We recruited a sample of 1938 nurses working in the selected hospitals through convenience sampling. The inclusion criteria of the study were as follows: 1) nurses who received a nursing license from a national legal institute; 2) nurses without communication difficulty with other nurses; and 3) nurses who agreed to participate in the present study. Nurse managers and head nurses were excluded from the study because being in a managerial position may significantly impact their work quality of life and these individuals do not provide an accurate representation of nurses in China. Sixty-five nurses from the first investigated division were re-measured two weeks later to assess the test-retest reliability of the scale.

### Instruments

#### The QNWL questionnaire

Work-related factors were measured by the QNWL questionnaire with the permission of Professor Brooks, who developed the original questionnaire using the O’Brien-Pallas and Baumann framework. The questionnaire consisted of 42 items and used a 6-point rating scale ranging from “strongly disagree” to “strongly agree” [16]. Scores were created by summing and averaging the items; high scores represented high levels of each construct. Negatively worded items. The QNWL tool includes the following 4 subscales: 1) work life-home life dimension, which consists of 7 items measuring the interaction between the nurse’s work and home life; 2) the work design dimension, which consists of 10 items measuring the composition of nursing work, such as work load, staffing, and autonomy; 3) the work context dimension, which consists of 20 items measuring the nurses’ work settings and the impact of the work environment on nurses and patients; and 4) the work world, which consists of 5 items measuring the effects of broad social influences and changes on nursing practice.

The QNWL questionnaire has been demonstrated to be a valid and reliable measurement tool for various aspects of nursing work life. According to Brooks, the QNWL’s test-retest reliability was 0.90, and the Cronbach’s α values were > 0.55 for each subscale [17].

A translation and back-translation process was employed in the pilot study prior to applying the questionnaire in China. Two experts in the same area of study, who spoke satisfactory English but were not familiar with the scale, were invited to translate the English version of the scale into Chinese. After comparison and discussion, a preliminary draft was obtained. The translation quality of the draft was evaluated by two English teachers and then was discussed by six professionals who are experienced in scale design from the fields of public health, clinical nursing, clinical care and psychology, to generate a revised version (S1 Table). Thirty nurses from some of the divisions of the hospital where the investigators work were selected randomly for retesting, and a final draft was obtained after modifications.

#### The WHOQOL-BREF

The WHOQOL-BREF has 26 items; it is a 5-point scale used to assess quality of life. The WHOQOL-BREF consists of four subscales (physiology domain, psychology domain, social relationship domain, and environmental domain), with good reliability and validity. The Chinese version of the WHOQOL-BREF was translated and modified by Fang Jiqian, and this questionnaire has been widely used in China with strong reliability and validity. In the present study, Cronbach’s α for the WHOQOL-BREF was 0.83.

### Procedure and ethical considerations

We explained the study purpose and procedures to the participating nurses. Voluntary participation and data confidentiality were clarified.

We performed a pilot study with 30 nurses to test the logistics of the study, and we did not find any problems from the nurses in understanding the QNWL items.

We also conducted several training sessions for the research assistant to ensure that the data collection process was consistently performed for all nurse participants.

### Data collection and analysis

After the research assistant received informed consent, the participants were asked to complete the demographic scale, the QNWL and the WHOQOL-BREF and then to return the completed scales to the research assistant. Scale completion required approximately 10–13 minutes.

A demographic scale was used to summarize demographic characteristics of the study sample. Cronbach’s α was used to assess the internal consistency reliability of the QNWL scale. The intra-class correlation coefficient (ICC) was used to assess the test-retest reliability, and an ICC greater than 0.70 indicated acceptable test-retest reliability over 2 weeks [18, 19].

The correlation between the total scores of the QNWL and WHOQOL-BREF was used to assess the criterion-relation validity of the QNWL scale, and a correlation coefficient between 0.4 and 0.8 was satisfactory [20].

Pearson’s correlation coefficients of each item of the QNWL scale with the four subscales and total score were used to assess the content validity of the scale. A higher correlation coefficient indicated better content validity [20].

The normal distribution of the total scores on the QNWL and WHOQOL-BREF was analyzed. When the scores did not satisfied a normal distribution, the Mann-Whitney test was used to compare between the groups, and the Kruskal-Wallis test was used among groups to assess the discrimination validity of the scale for different populations.

A factor model was constructed according to the theoretical structure of the QNWL. Confirmatory factor analysis was performed, and the theoretical factor model was fit with actual data. The factor loading values indicated that the items had significant loadings on the four subscales. Factor loads greater than 0.3 indicated subordinate relationships and a strong correlation between the item and the corresponding subscale [21], as well as the existence of the theoretical structure of the QNWL. The construct validity of the scale was assessed using the following indices: χ2 statistics and degrees of freedom for the overall fit of the model to data, the adjusted goodness of fit index, (AGFI), the relative mean square error of approximation (RMSEA), the standardized root mean square residual (SRMR), the Akaike information criterion (AIC), the consistent Akaike information criterion (CAIC), the comparative fit index (CFI), the normed fit index (NFI), the non-normed fit index (NNFI), and the incremental fit index (IFI). Statistical analysis was performed with SPSS software, version 16.0 (Chicago, IL, USA), and LISREL software, version 8.7 (Lincolnwood, IL, USA).

## Results

### Sample characteristics

A total of 1938 surveys were administered to nurses in the selected hospitals using a convenience sample. Of the 1922 nurses who completed questionnaires, we excluded those who did not provide complete data for the key outcome variables, including the QNWL and the WHOQOL-BREF. In total, 1922 nurses provided the final data used for the analyses. The overall response rate was 99.17% (n = 1922). The typical characteristics of the 1922 were 96.1% female (n = 1847), average age of 30.0±6.93 (ranging 19–55) years old, 54.4% (n = 1045) married and an average working time of 9.0±7.7 (ranging 0.5–38) years. The typical respondents were 82.7% staff nurses (n = 1590), 13.7% team leaders (n = 263) and 3.6% assistant nurses (n = 69). Overall, 38.3% (n = 736) of the 1922 respondents were working full time in internal medicine, 25.2% (n = 485) in surgery, 22.1% (n = 426) in emergency and ICUs, and 3.7% (n = 71) in obstetrics and gynecology (Table 1).

**Table 1:** The sociodemographic characteristics of the study sample (n = 1922).

Characteristic	Means ± SD
Age	30.0±6.93
Working time	9.0±7.7
Characteristic	n (%)
Sex	
Female	1847 (96.1%)
Male	75 (3.9%)
Marital status	
Married	1045 (54.4%)
Single	849 (44.2%)
Other	28 (1.4%)
Status	
Junior nurses	846 (44.0%)
Senior nurses	685 (35.6%)
Nurses in charge	391 (20.3%)
Nursing position	
Staff nurses	1590 (82.7%)
Team leader	263 (13.7%)
Assistant nurses	69 (3.6%)
Working department	
Internal medicine	736 (38.3%)
Surgery	485 (25.2%)
Emergency and ICU	426 (22.1%)
Pediatrics	106 (5.5%)
Obstetrics and gynecology	71 (3.7%)
Others	98 (5.2%)
Personal income (monthly)	
<1500 Yuan	123 (6.4%)
1500 Yuan -	133 (6.9%)
3000 Yuan -	982 (51.1%)
5000 Yuan-	684 (35.6%)
Age of children	
No children	1015 (52.8%)
Younger than 3 years old	225 (11.7%)
3 years-5 years old	160 (8.3%)
6 years-17 years old	405 (21.1%)
18 years old and older	117 (6.1%)

### Internal consistency reliability

The Cronbach’s α of the entire QNWL scale was 0.912; the values were 0.588, 0.574, 0.655, and 0.622 for the subscales measuring work design, work life-home life, work context, and work world, respectively. The subscale-to-total scale correlates were 0.697 (p<0.01) for work design, 0.716 (p<0.01) for work life-home life, 0.946 (p<0.01) for work context, and 0.717 (p<0.01) for work world (Table 2). The correlation coefficients between the subscales were 0.65 (p<0.01) between work life-home life and work context, 0.74 (p<0.01) between work life-home life and work design, 0.77 (p<0.01) between work life-home life and work world, 0.83 (p<0.01) between work context and work design, 0.82 (p<0.01) between work context and work world, 0.73 (p<0.01) between work design and work world.

**Table 2:** The internal consistency reliability and criterion-relation validity of the QNWLS.

Observable Variable	Cronbach's α value	QNWLS total scores	WHOQOL-BREF total scores	Factor loading	T value	R^2^
Item 1	0.909	0.490**	0.486**	0.52	22.84	0.27
Item 2	0.908	0.592**	0.479**	0.66	30.44	0.44
Item 3	0.916	-0.183**	-0.198**	-0.30	-12.53	0.09
Item 4	0.908	0.593**	0.395**	0.67	28.90	0.46
Item 5	0.908	0.596**	0.514**	0.68	31.85	0.46
Item 6	0.910	0.450**	0.257**	0.41	17.18	0.17
Item 7	0.908	0.604**	0.359**	0.61	28.68	0.37
Item 8	0.909	0.560**	0.327**	0.55	25.07	0.30
Item 9	0.909	0.566**	0.315**	0.58	26.83	0.33
Item 10	0.909	0.532**	0.395**	0.71	33.38	0.50
Item 11	0.917	-0.062**	-0.124**	-0.20	-8.00	0.04
Item 12	0.909	0.558**	0.460**	0.71	33.44	0.50
Item 13	0.911	0.324**	0.205**	0.30	12.99	0.09
Item 14	0.907	0.631**	0.365**	0.64	30.38	0.41
Item 15	0.909	0.551**	0.311**	0.57	26.49	0.33
Item 16	0.916	-0.084**	-0.129**	-0.25	-10.15	0.06
Item 17	0.909	0.559**	0.388**	0.64	28.88	0.40
Item 18	0.910	0.495**	0.279**	0.56	24.62	0.31
Item 19	0.908	0.597**	0.349**	0.61	28.94	0.38
Item 20	0.917	-0.166**	-0.154**	-0.27	-11.28	0.07
Item 21	0.908	0.594**	0.271**	0.59	27.39	0.34
Item 22	0.909	0.546**	0.306**	0.56	25.69	0.31
Item 23	0.910	0.471**	0.270**	0.46	19.77	0.21
Item 24	0.909	0.541**	0.394**	0.61	26.21	0.38
Item 25	0.909	0.533**	0.374**	0.57	25.52	0.33
Item 26	0.908	0.569**	0.306**	0.57	26.50	0.33
Item 27	0.909	0.499**	0.331**	0.68	31.62	0.46
Item 28	0.908	0.588**	0.368**	0.59	27.72	0.35
Item 29	0.909	0.550**	0.327**	0.55	25.34	0.30
Item 30	0.909	0.518**	0.282**	0.52	23.61	0.27
Item 31	0.908	0.594**	0.329**	0.66	28.06	0.36
Item 32	0.909	0.570**	0.346**	0.58	26.96	0.34
Item 33	0.908	0.642**	0.373**	0.68	32.74	0.46
Item 34	0.907	0.676**	0.389**	0.71	35.28	0.51
Item 35	0.908	0.561**	0.322**	0.58	26.72	0.33
Item 36	0.909	0.507**	0.320**	0.67	30.98	0.44
Item 37	0.913	0.202**	0.117**	0.12	4.64	0.01
Item 38	0.908	0.590**	0.342**	0.58	26.93	0.34
Item 39	0.911	0.336**	0.204**	0.40	16.36	0.16
Item 40	0.907	0.633**	0.372**	0.64	30.58	0.41
Item 41	0.912	0.219**	0.129**	0.09	3.61	0.01
Item 42	0.910	0.460**	0.262**	0.46	19.65	0.21
Work design	0.588	0.697**	0.524**			
Work life/home life	0.574	0.716**	0.400**			
Work context	0.655	0.946**	0.544**			
Work world	0.622	0.717**	0.475**			
QNWLS total scores			0.605**			

** p<0.01.

### Test-retest reliability

The ICC was 0.74, indicating high test-retest reliability of the QNWL questionnaire over a 2-week period. The ICCs was 0.75, 0.78, 0.93, and 0.68 for the work life-home life, work design, work context, and work world subscales, respectively.

### Criterion-relation validity

The criterion-relation validity of QNWL was acceptable. The correlation coefficient between the QNWL total score and the WHOQOL-BREF total score was 0.605 (p<0.01) (Table 2).

### Content validity

**Fig 1:**
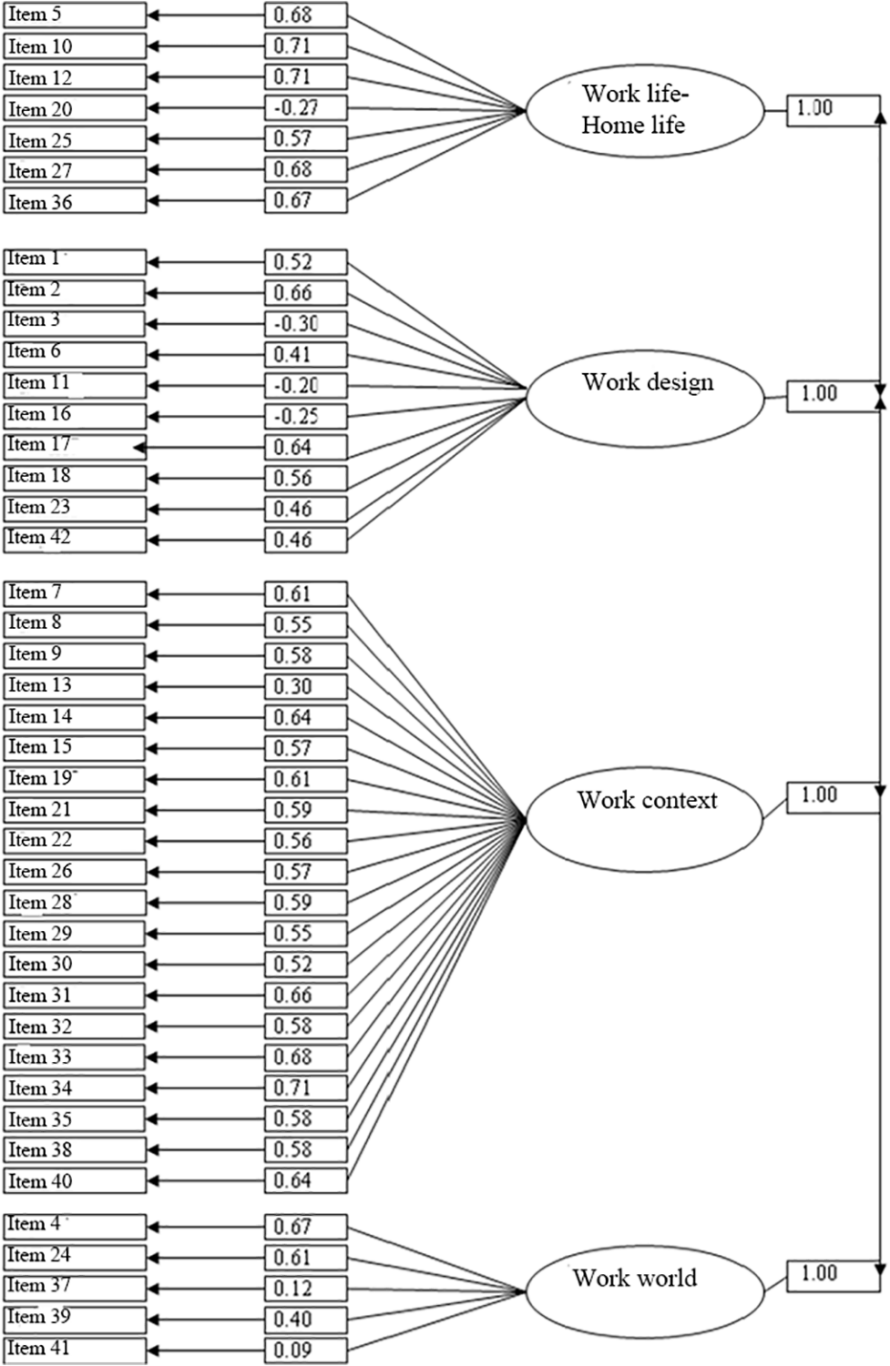
Confirmatory factor analysis of the QNWL. χ2 = 13879.60, df = 813, p = 0.0001, RMSEA = 0.091, AIC = 1806.00, CAIC = 7730.69, SRMR = 0.072, CFI = 0.93, GFI = 0.74, AGFI = 0.72, NFI = 0.92, NNFI = 0.92, IFI = 0.93.

The Pearson’s correlation coefficients of items 3, 11, 16, 20, 37, and 41 with the QNWL total score were -0.183, -0.062, -0.084, -0.166, 0.202, and 0.219, respectively (Table 2). At the same time, the factor loads of these items to their corresponding subscales were low (Table 2 and Fig 1). These results suggested that these items failed to accurately measure what QNWL was expected to measure and therefore should be removed or modified.

### Discrimination validity

The QNWL scale showed good discrimination validity for different nurse populations. The total scores showed significant differences in marital status, nursing position, personal income (monthly), ages of children, working time, and age of the nurse (p<0.05) (Table 3).

**Table 3:** The discrimination validity of the QNWLS and WHOQOL-BREF.

Characteristic	n (%)	WHOQOL-BREF total scores	QNWLS total scores
**Sex**			
Male	75 (3.9%)	76.71±14.21	160.81±24.87
Female	1847 (96.1%)	76.97±11.88	157.96±22.25
Z value		-0.008	-1.307
P value		0.993	0.191
**Marital status**			
Married	1045 (54.4%)	76.67±11.68	159.34±21.53
Single	849 (44.2%)	77.16±12.21	156.52±22.86
Separated	4 (0.2%)	81.47±9.65	160.00±26.89
Divorced	24 (1.2%)	76.63±11.58	164.17±21.85
F value		4.367	16.898
P value		0.359	0.002
**Aptitude**			
Junior nurses	846 (44.0%)	77.63±11.55	160.76±20.87
Senior nurses	685 (35.6%)	75.45±12.14	154.54±23.24
Nurses in charge	391 (20.3%)	78.13±12.36	158.45±23.07
F value		19.236	26.877
P value		0.001	0.001
**Nursing position**			
Staff nurses	1590 (82.7%)	76.38±11.89	157.09±22.28
Team leader	263 (13.7%)	80.11±12.50	163.15±22.36
Assistant nurses	69 (3.6%)	78.26±10.06	161.20±21.55
F value		20.576	18.858
P value		0.001	0.001
**Working department**			
Internal medicine	736 (38.3%)	76.54±12.25	157.29±22.47
Surgery	485 (25.2%)	77.37±11.51	157.82±21.80
Emergency and ICU	426 (22.1%)	76.38±12.40	158.03±22.55
Pediatrics	106 (5.5%)	78.77±12.17	159.67±23.62
Obstetrics and gynecology	71 (3.7%)	76.86±11.59	159.10±26.22
Other	98 (5.2%)	78.59±11.44	163.61±18.60
F value		3.908	4.673
P value		0.563	0.457
**Personal income (monthly)**			
<1500 Yuan	123 (6.4%)	79.21±11.21	165.73±20.72
1500 Yuan	133 (6.9%)	73.31±13.41	154.11±25.88
3000 Yuan	982 (51.1%)	76.05±11.64	156.87±21.63
5000 Yuan	684 (35.6%)	78.43±11.97	158.58±22.58
F value		31.208	20.761
P value		0.001	0.001
**Age of children**			
No children	1015 (52.8%)	76.73±11.48	159.46±21.23
Younger than 3 years old	225 (11.7%)	77.30±12.71	155.98±23.53
3 years-5 years old	160 (8.3%)	76.10±13.01	154.78±24.22
6 years-17 years old	405 (21.1%)	77.43±11.96	157.30±26.83
18 years old and older	117 (6.1%)	77.60±13.66	156.28±26.83
F value		3.943	10.911
P value		0.414	0.028
**Working time**			
Less 5 years	829 (43.1%)	77.30±11.54	160.84±21.27
5 years-9 years	431 (22.4%)	76.15±12.04	154.57±22.31
10 years-14 years	232 (12.1%)	76.69±12.17	155.83±23.55
15 years-19 years	161 (8.4%)	77.69±12.90	157.43±23.87
20 years or more	261 (13.6%)	77.25±12.37	157.97±22.63
F value		4.539	26.129
P value		0.338	0.001
**Age of nurses**			
Younger than 25 years old	614 (31.9%)	77.99±11.01	162.37±20.51
25 years-34 years old	912 (47.5%)	76.16±12.25	155.34±22.73
35 years-44 years old	316 (16.4%)	77.11±12.58	157.52±23.16
45 years old or older	80 (4.2%)	77.43±13.10	158.29±23.75
F value		11.391	36.359
P value		0.010	0.001

### Construct validity

The instrument consisted of 42 items. The first step of the CFA indicated that the matrix was suitable for factor analysis because Bartlett's test of sphericity was P*<*0.0005, and the Kaiser-Meyer-Olkin measurement of sampling accuracy was 0.93.

The factor loads of Item 20 were -0.27 for the work life-home life subscales, The factor loads of Item 3, Item 11 and Item 16 were -0.30, -0.20, -0.25, respectively, for the work design subscales. The factor loads of Item 13 were 0.30 for the work context subscales. The factor loads of Item 37 and Item 41 were 0.12 and 0.09, respectively, for the work world subscales (Fig 1). These results suggest that Items 3, 11, 13, 16, 20, 37, and 41 cannot accurately reflect what the QNWL scale is expected to measure and that the correlations of these items with the corresponding subscales are low. The chi-square test (χ2 = 13879.60 df (degree of freedom) = 813, p = 0.0001) was significant. The RMSEA value was 0.091, and SRMR = 0.072. Other fit indices included AIC = 1806.00, CAIC = 7730.69, CFI = 0.93, GFI = 0.74, AGFI = 0.72, NFI = 0.92, NNFI = 0.92, and IFI = 0.93 (Fig 1).

## Discussion

The original version of the QNWL is in English. Linguistic or cultural biases or restrictions would consequently limit its utility without translation. Therefore, we translated the scale using Brislin’s forward-backward translation model [22]. The translation processes in the research were performed competently so that the final version was reliable.

The QNWL total scale has acceptable internal consistency with a Cronbach’s *α* value of 0.912, exceeding the criteria. However, Item 20 “I feel that rotating schedules negatively affect my life”, Item 3 “My workload is too heavy”, Item 11 “I perform many non-nursing tasks”, Item 16 “I experience many interruptions in my daily work routine”, Item 13 “Friendships with my co-workers are important to me”, Item 37 “I would be able to find the same job in another organization with about the same salary and benefits”, and Item 41 “I believe my work impacts the lives of patients/families” all had factor loads <0.30. Presumably, this result should be ascribed to cultural differences, which caused the low contributions of these items to the QNWL. In the future, the details of the items of the QNWL should be further discussed and improved to ensure the accuracy of data assessment.

The four subscales correlated strongly with each other, indicating a satisfactory degree of homogeneity among all of the subscales. With regard to the test-retest reliability, the high ICCs for the total scale and subscales demonstrated strong stability of the QNWL over time. For construct validity, the CFA results demonstrated a good fit for the QNWL. However, three items of the QNWL did not meet the minimum acceptable factor loading criterion. These items included “I feel that rotating schedules negatively affect my life”, “I believe my work impacts the lives of patients/families” and “I would be able to find the same job in another organization with about the same salary and benefits.” Following another round of expert review, we think these items should be kept in the scale.

Table 3 shows that both the QNWL and WHOQOL-BREF had satisfactory discrimination validity. Furthermore, the discrimination validity of the QNWL showed statistical significance in marital status, age of children, and working lifetime. These findings indicated that these factors had noticeable influences on the working status of nurses, although they may not have had significant influences on overall quality of life for other professions. Our results were consistent with the working status of nurses commonly seen in clinical practice.

Work design results indicated nurses endure more stress from work, leading to exhaustion. This stress can be related to the high workload and severe nurse shortages. According to the World Health Report 2006, the average density of nurses per 1,000 inhabitants throughout the world is 4.06, whereas the density of nurses per 1,000 inhabitants in China is only 1.06 [13]. Moreover, according to a report from China, the nurse turnover rate in Shanghai from 2001 to 2005 was 12.8% [23]. This finding suggests the critical situation in the retention of the existing nursing workforce in China. According to previous studies available from China, low pay and high workload were the main reasons reported for nurse turnover [24, 25], which is consistent with the results of our study. Nurses in China frequently suffer from occupational stress owing to a higher demand for better medical services and a more complex nurse-patient relationship [26]. Work context results indicated that nurse managers in mainland China should focus on improving the nursing environment. Western countries became aware of the importance of developing healthy environments for nurses earlier than mainland China. In the 1980s, the American Nurses Association (ANA) developed a magnet hospital focusing on creating healthy work environments, developing an organizational culture that builds on respect, valuing individuals, and striving for higher quality in service and outcomes. Currently, Western countries are taking a series of measures to build a healthy work environment. However, mainland China appears to pay less attention to developing or optimizing the nursing work environment; based on the low availability of published articles, minimal research focused on exploring methods to develop a satisfactory work environment has been conducted. The work world results indicated most nurses in China were not satisfied with their pay, which is largely related to the nurses’ work department. Therefore, an imbalance between effort and reward, which can influence a nurse’s job satisfaction, exists. According to Zeng, one of the specific stressors among Chinese nurses was effort–reward imbalance [27]. Therefore, nurse managers should offer nurses more career promotion opportunities and social support and increase pay. These indications mentioned above were limited to the first testing of the QNWL tool with a convenience sample in south China.

The strengths of this study included its large sample size from a multi-level hospital in China. However, the participants were all selected from tertiary hospitals. Thus, the generalization of the results must be limited. To determine more accurately nurses’ QNWL in the investigated area, the psychometric properties of the QNWL should be explored in lower-level hospitals. To achieve a more reasonable study design, stratified sampling should be employed.

The QNWL shows promise for use as a measurement tool of QNWL among nurses in Mainland China. Although additional modifications to the tool are needed, it may be possible to use the QNWL in hospitals to understand the work life and work environment of staff nurses. A better understanding of QNWL is fundamental to the specific strategies aimed at improving QNWL and organizational productivity. The effectiveness of specific strategies, including nurses’ participation in decision-making, removing non-nursing tasks, and building healthy environments, could be evaluated with this instrument. The achievement of greater QNWL may increase nurses’ job satisfaction and improve patient care as well as organizational productivity.

## Supporting Information

S1 Table Instrument items of the QNWL. (DOC)
