# Supplementary material for: Validation of the Chinese Version of the Quality of Nursing Work Life Scale
Source: PLoS One. 2015 May 7;10(5):e0121150. doi: 10.1371/journal.pone.0121150 (PMC4423946; doi:10.1371/journal.pone.0121150)
Supplement: S1 Table — (DOC) [file pone.0121150.s001.doc]

**S1 Table. Instrument items of the QNWL.**

| 1. I receive a sufficient amount of assistance from unlicensed support personnel. |  | | | | | | | | |
| --- | --- | --- | --- | --- | --- | --- | --- | --- | --- |
| 2. I am satisfied with my job. | |  | |  | | |  | | |
| 3. My workload is too heavy. | |  | |  | | |  | | |
| 4. I believe that, in general, society has the correct image of nurses. | | | | | | | |  | |
| 5. I am able to balance my work with my family needs. | | | | |  | | | |  |
| 6. I have autonomy to make patient care decisions. | | | | |  | | | |  |
| 7. I am able to communicate well with my nurse manager/supervisor. | | | | | | | |  | |
| 8. I have adequate patient care supplies and equipment. | | | | |  | | | |  |
| 9. My nurse manager/supervisor provides adequate supervision. | | | | | | | |  | |
| 10. I am able to arrange for child -care when I am at work. | | | | |  | | | |  |
| 11. I perform many non-nursing tasks. | | |  | | |  | | | |
| 12. I have energy left remaining after work. | |  | |  | | |  | | |
| 13. Friendships with my co-workers are important to me. | | | | |  | | | |  |
| 14. My work setting provides career advancement opportunities | | | | | | | |  | |
| 15. I feel like there is teamwork in my work setting. | | | | |  | | | |  |
| 16. I experience many interruptions in my daily work routine. | | | | |  | | | |  |
| 17. I have enough sufficient time to do my job well. | | |  | | |  | | | |
| 18. There are enough a sufficient number of RNs in my work setting. | | |  | | |  | | | |
| 19. I feel like I belong to a “work family”. | | |  | | |  | | | |
| 20. I feel that rotating schedules negatively affect my life. | | | | |  | | | |  |
| 21. I am able to communicate with other therapists (physical, respiratory, etc.). | | | | | | | |  | |
| 22. I receive feedback on my performance from my nurse manager/supervisor. | | | | | | | |  | |
| 23. I am able to provide good quality patient care. | | |  | | |  | | | |
| 24. My salary is adequate for my job given the current job market conditions. | | | | | | | |  | |
| 25. My organization’s policy for family-leave time is adequate. | | | | |  | | | |  |
| 26. I am able to participate in the decisions made by my nurse manager/supervisor. | | | | | | | |  | |
| 27. I am able to arrange for day care for my elderly parents. | | | | |  | | | |  |
| 28. I feel respected by the physicians in my work setting. | | | | |  | | | |  |
| 29. The nurses’ lounge/break-area/locker room in my setting is comfortable. | | | | | | | |  | |
| 30. I have access to degree completion programs through my work setting. | | | | | | | |  | |
| 31. I receive support to attend in-services and continuing education programs. | | | | | | | |  | |
| 32. I communicate well with the physicians in my work setting. | | | | | | | |  | |
| 33. I am recognized for my accomplishments by my nurse manager/supervisor. | | | | | | | |  | |
| 34. Nursing policies and procedures facilitate my work. | | | | |  | | | |  |
| 35. I feel that the security department provides a secure environment. | | | | | | | |  | |
| 36. I am able to arrange for day care when my child is ill. | | | | |  | | | |  |
| 37. I would be able to find the same job in another organization with about the same salary and benefits. | | | | | | | |  | |
| 38. I feel safe from personal harm (physical, emotional, or verbal) at work. | | | | | | | |  | |
| 39. I feel that my job is secure. | |  | |  | | |  | | |
| 40. I feel that the upper-level management has respect for nursing. | | | | | | | |  | |
| 41. I believe that my work impacts the lives of patients/families. | | | | |  | | | |  |
| 42. I receive quality assistance from unlicensed support personnel. | | | | | | | |  | |
